# Epilepsy Successfully Treated with Nitrate of Silver, Etc.

**Published:** 1848-04

**Authors:** Charles D. Hendry


*Epilepsy successfully treated with the Nitrate of Silver, and an Antispasmodic Powder composed of sage, ginger, and mustard.* By Charles D. Hendry, M.D.—Of all the diseases to which the human frame is subject, no one is more alarming in its symptoms, or more fatal in its effects, than epilepsy. It is a disease of the nervous system, located in the brain, and exclusively chronic in its character, attacking persons of all ages, from childhood to old age, without regard to constitution. Often times its victims are among the most robust, but more frequently the emaciated. So far as my experience will warrant an opinion, those with impaired constitutions, are more liable to suffer from this malady. Viewing epilepsy as a chronic disease from its commencement, and having been baffled in the treatment which had been confined to the paroxysms, for which antiphlogistic remedies have invariably been resorted to, I resolved upon an opposite course, and have employed tonics and stimulants. For several years I attended Judge R., of Burlington county, for epilepsy, aged fifty years; of plethoric habit, and sanguine temperament. The treatment in his case had been confined to the paroxysms, for which copious depletion with the lancet had invariably been resorted to without benefit, the attacks returning more frequently and severely. Having lost all confidence in the course adopted, I resolved upon the opposite,—a stimulating treatment. The premonitory symptoms, such as stupor and difficult deglutition, were always prominent some hours before the disease would be fully developed. In this stage I advised the free use of brandy in warm water, to be given as frequently as circumstances required. The disposition to epilepsy soon disappeared, and he lived several years, and enjoyed uninterrupted health. The principal cause of epilepsy, I consider not to depend upon congestion,—from a predisposition of blood to the brain, but to debility, giving rise to paralysis, and as corroborative evidence of this theory, I will cite several prominent cases, of some twenty, treated successfully, without an exception, by a tonic treatment. All of them had been subjected to an antiphlogistic course, without relief. A great deal depends upon the perseverance of the physician, as there is always a strong tendency in the disease to return. By pursuing this course, I am confident a large majority of the epileptics of the country will be banished from the incurable lists of physicians, and relieve them of the unpleasant task of so often pronouncing them beyond the reach of professional aid. The great mistake in the treatment of epilepsy, has in a measure been owing to its having been confined to the paroxysms, when in reality the intermission is the time for action, bracing the system with the most active tonics and antispasmodics, and in this way producing a radical change in the nervous system. As I have no disposition to occupy the columns of the journal unnecessarily, I will be content by citing five prominent cases, which I trust will suffice to give countenance to the treatment proposed.

Case I.—Mr. J. J. of Gloucester county, by occupation a farmer, aged fifty years, of full habit, general health impaired. He had been subject to epilepsy for three years, the attacks recurring every six weeks, and varying from one to three convulsions. His family physician at sundry times resorted to copious depletion, both general and local, cathartics, emetics, revellants, &c., but all to no purpose, and finally abandoned his case as incurrable. In the month of March, 1845, I commenced the treatment of his case with one-fourth grain doses of the nitrate of silver, in form of pill, three times a day, in a teaspoonful of the antispasmodic powder, mixed in an equal quantity of molasses. This course was continued three months, at which time his disease had assumed a more favorable aspect. Half grain doses of the pill was now advised, and a gradual increase of the dose of the powder; at the expiration of six months the disease left him, and now two years have elapsed and no return of the disease. It was not thought advisable to make any change in his diet, except to abstain from the free use of coffee.

Case II.—Mr. A. D. of Camden county, aged 35 years, with constitution much impaired, and emaciated, complexion sallow, and imbecile in mind; he had been afflicted with epilepsy from childhood, and was subjected to medical treatment in early life whilst he resided in Burlington county, but without benefit. For a number of years his case had been considered hopeless, the paroxysms, would return every six weeks and continue irregularly for several days. During this period he would have from ten to fifty convulsions, the attack resulting in a state of decided mania. In December, 1844, his friends made application to me respecting his case. The treatment advised was so similar to the preceding case that I will not recapitulate it, but will merely add that in less than three months I had every assurance that the treatment recommended would ultimately effect a cure, and in less than nine months I had the satisfaction to see him relieved of this formidable disease, his constitution reacted, his general health was restored, and I am happy to state there is no appearance of imbecility of mind. Several years have now transpired and his health continues good.

Case III.—The subject of this case was a daughter of Mr. C., of Camden county, aged 3 years. She had been afflicted with epilepsy for six months, constitution impaired; during this period she had more than one hundred distinct epileptic convulsions; primary cause supposed to be gastric irritation. I had charge of this case from the commencement, and resorted to topical blood-letting, with leeches, cathartics, emetics, revellants, and a variety of antispasmodics, but all without the desired effect. After six months' treatment the parents became discouraged, and removed the child to an adjacent county for the purpose of obtaining the advice of several practitioners, who agreed that the patient was idiotic, and any medical treatment would prove of no advantage. On their return I was again called upon to prescribe in her case, and recommended the pill and powder in the accustomed dose without regard to age, and in less than three months the disease left her, and she is now one of the most intelligent and interesting children in the village in which she resides. Eighteen months have elapsed and no return of the disease.

Case IV.—Mrs. W. of Camden county, aged 40 years, of plethoric habit and in the enjoyment of good health, and now in the fourth month of utero gestation. When called to her she informed me she was the mother of four children, and during each term, and about the fifth month, she would be assailed with convulsions, which returned regularly every two weeks during the remainder of the term. She had been subjected to medical treatment in Philadelphia, which was confined to copious depletion with the lancet, counter-irritants, and enemas, but all to no purpose, the convulsions continuing at intervals during each term of pregnancy. About the fifth month I was summoned to visit her, and found her struggling with a frightful convulsion. I was entreated by her friends to resort to venesection, but as it had on all previous occasions proved of no avail, I objected, and recommended counter-irritants and enemas. After the paroxysm I advised the pill and powder in the ordinary dose. She had not a return of the disease, but passed through the term without inconvenience. This is the first case, under such circumstances, in which I have had an opportunity of testing the utility of this form of treatment.

Case V.—In October, 1846, I was requested to visit the daughter of Mr. J. S. T., of Burlington county, aged five years; emaciated and afflicted with chorea and epilepsy; the former disease evidently the existing cause of the latter. The history of her case I will briefly state. When about two years old she was assailed with epilepsy, which returned every night for some two years, with but one or two intermissions of four weeks, and on one occasion, a short time previous to my seeing her, she remained in a state of stupor for nine days, the result of an attack of epilepsy. Her parents informed me she had been under treatment for a long time, but was finally abandoned as incurable. When I first visited her she was restless beyond description, requiring some one to watch her constantly, and I supposed her intellect to be completely destroyed, and prescribed for her with great reluctance; however, in the course of four weeks I completely removed the chorea with equal parts of the bi tart. potass. and flor. sulph., in teaspoonful doses, mixed in molasses, three times a day. I then ordered the pill and powder in the accustomed doses, and in six months was assured by her parents that she was perfectly well. The last attack was in April, 1847.—*New Jersey Med. Reporter.*

